# A proposal to explain “covert” increases in intracranial pressure

**DOI:** 10.1186/2045-8118-12-S1-P16

**Published:** 2015-09-18

**Authors:** Doug Hamilton, Mark Hamilton, Alim Mitha, John Tyberg

**Affiliations:** 1 University of Calgary, Calgary, Canada

## Introduction

Some substantial number of unfortunate patients suffer from the symptoms and signs of elevated cerebrospinal fluid (CSF) pressure, even though pressure measured in the cerebral venous system is normal. We hypothesize that, with elevated CSF pressure, cerebral microvascular congestion may occur, even though (subdural) large-vein pressure has been shown to be normal.

## Background

Even with elevated CSF pressure, large-vein pressure could be normal because the large veins are “tented” open by their structural relation to the dura. However, the smaller, penetrating veins are not protected by the dura in this way so they could collapse. By this “Starling-resistor” action, venular and microvascular pressure would have to increase if flow were to continue. This increased microvascular pressure might be measured by an adaptation of cardiologists’ wedge-pressure logic. (When an artery is obstructed by a catheter or by the inflation of a balloon, the pressure measured in the stationary column of blood beyond the obstruction is equal to the downstream pressure.)

## Hypothesis

These concepts are illustrated in the figure. Solid lines indicate the relationships of intravascular pressure when CSF pressure is normal (~10 mmHg). Dashed lines indicate the relationships when CSF pressure is elevated (~35 mmHg). If flow is to continue, the pressure in the capillaries and in the small penetrating veins must exceed CSF pressure. However, pressure in the large “dura-strutted” veins will continue to be normal and to be governed only by right atrial pressure.

## Conclusion

Experimental verification of this hypothesized mechanism has been impeded by the presence of an arterial rete in many species other than in dogs or in human subjects and dogs have been unavailable to us. Even in the absence of experimental verification, we suggest that these concepts have important therapeutic implications: normal dural vein pressure should NOT rule out elevated CSF pressure and alternative measures of CSF pressure should be employed.

## Consent to publish

Written informated consent for publication of their clinical details was obtained from the patient.

